# Concurrent Intramuscular Thigh Hematoma and Femoropopliteal Venous Thrombosis During Supratherapeutic Warfarin Anticoagulation

**DOI:** 10.3390/diagnostics16183002

**Published:** 2026-09-16

**Authors:** Hung-Ming Lee, Tse-Hao Chen

**Affiliations:** 1 Department of Emergency Medicine, Mackay Memorial Hospital, Taipei 104217, Taiwan; 2 Graduate Institute of Injury Prevention and Control, College of Public Health, Taipei Medical University, Taipei 11031, Taiwan

**Keywords:** warfarin, intramuscular hematoma, femoropopliteal venous thrombosis, CT, Doppler ultrasonography

## Abstract

An anticoagulated patient with unilateral thigh swelling may have more than one process. A 79-year-old man receiving long-term warfarin for atrial fibrillation and a previous ischemic stroke presented with 3 days of atraumatic left-thigh pain, swelling, and inability to walk. His international normalized ratio was 6.41. Computed tomography (CT) showed an approximately 4 cm × 7 cm intramuscular hematoma in the left vastus intermedius. Duplex ultrasonography additionally demonstrated femoropopliteal venous thrombosis causing partial occlusion, with absent spectral flow in the sampled femoral vein segment and residual popliteal venous flow. Warfarin was initially withheld without reversal therapy. CT-guided drainage was performed after the international normalized ratio decreased to 1.44, and follow-up CT angiography showed no active bleeding. Edoxaban 60 mg once daily was started 8 days after drainage. Following rehabilitation, the patient was discharged in stable condition and was able to walk independently. These images illustrate that hemorrhage and venous thrombosis can coexist despite supratherapeutic anticoagulation; CT and duplex ultrasonography provide complementary information that can prevent diagnostic anchoring and guide the sequence of source control and anticoagulation resumption.

**Figure 1:**
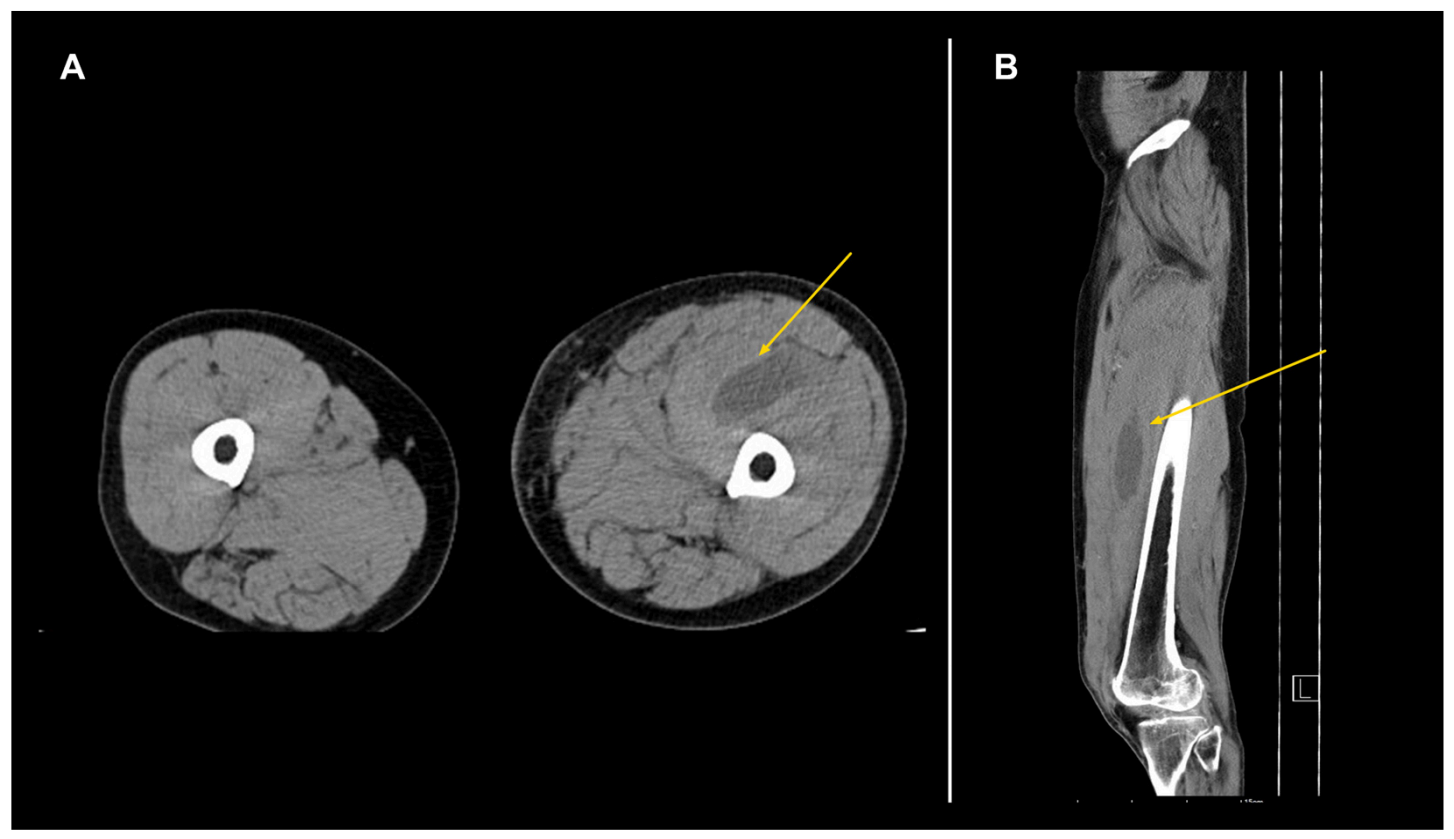
Computed tomography (CT) findings of an atraumatic left-thigh intramuscular hematoma during supratherapeutic warfarin anticoagulation. A 79-year-old man with hypertension, atrial fibrillation, and a previous ischemic stroke had received warfarin for 6 years, with historical international normalized ratio (INR) values of 2.0–3.0. He presented with 3 days of progressive left-thigh pain and swelling severe enough to prevent walking. He reported no trauma, and there was no visible wound, erythema, pallor, or cyanosis. His blood pressure was 129/84 mmHg, heart rate was 79 beats/min in atrial fibrillation, prothrombin time was 61.3 s (control, 11 s), and INR was 6.41. Axial (**A**) and sagittal (**B**) CT images show an approximately 4 cm × 7 cm intramuscular hypodense collection in the left vastus intermedius, compatible with a hematoma (yellow arrows). In a multicenter cohort of 375 patients with major muscular hematoma associated with antithrombotic therapy, the lower limbs were the most frequent site, and in-hospital mortality was 8.5% [1]. A 2026 retrospective study of 219 anticoagulated patients with spontaneous abdominal or pelvic soft-tissue hematomas found that hemodynamic instability, larger hematoma volume, arterial-phase contrast extravasation, and fascial rupture were more frequent among patients selected for transarterial embolization [2]. Warfarin was initially withheld without reversal therapy. On hospital day 4, after the INR had decreased to 1.44, CT-guided catheter drainage yielded dark-red fluid; blood and aspirate cultures showed no growth. The panels were cropped from de-identified source images, juxtaposed, and labeled; arrows were added only to localize the hematoma. No local enhancement or filtering was applied.

**Figure 2:**
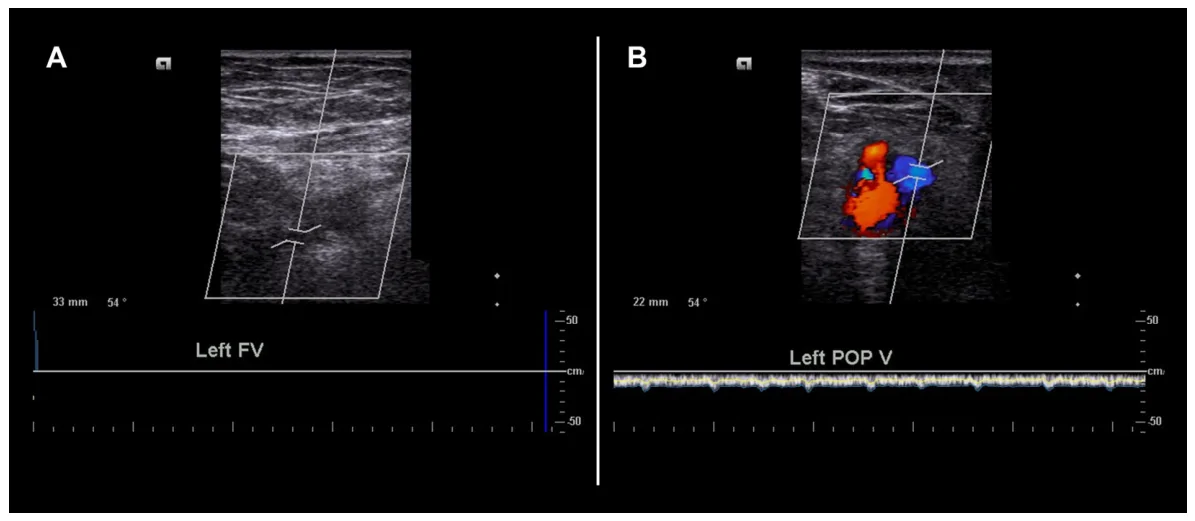
Duplex ultrasonography findings of concomitant left femoropopliteal venous thrombosis. Because venous obstruction remained in the differential diagnosis of unilateral limb swelling, a complete lower-extremity duplex examination was performed. (**A**) No detectable color or spectral Doppler flow was recorded in the femoral vein segment. (**B**) Residual color and spectral Doppler flow was present in the popliteal vein segment. The complete examination demonstrated femoropopliteal venous thrombosis causing partial occlusion. FV, femoral vein; POP V, popliteal vein. The patient had no previous history of deep venous thrombosis or pulmonary embolism, and no previous venous ultrasonographic examination was available for comparison. Venous thrombosis detected during anticoagulant therapy warrants evaluation for potential contributing factors [3]. Follow-up CT angiography on hospital day 12 showed no active bleeding. Expert consensus recommends reassessing the need for anticoagulation after bleeding is controlled and restarting it when medically safe in patients who are not at low thromboembolic risk [4]. Edoxaban 60 mg once daily was initiated 8 days after drainage. In a randomized, double-blind trial involving 21,105 patients with moderate-to-high-risk atrial fibrillation, once-daily edoxaban was noninferior to warfarin for preventing stroke or systemic embolism and was associated with a lower rate of major bleeding [5]. Following rehabilitation, the patient was able to walk independently at discharge. The complementary use of CT and duplex ultrasonography prevented diagnostic anchoring on the hematoma and documented two clinically relevant processes requiring sequential management.

## Data Availability

The de-identified data and original images supporting this report are available from the corresponding author upon reasonable request. They are not publicly available to protect patient privacy.
